# Factors Associated with (Exclusive) Breastfeeding Duration—Results of the SUKIE-Study

**DOI:** 10.3390/nu14091704

**Published:** 2022-04-20

**Authors:** Bernadette Bürger, Karin Schindler, Tanja Tripolt, Antonia Griesbacher, Hans Peter Stüger, Karl-Heinz Wagner, Adelheid Weber, Alexandra Wolf-Spitzer

**Affiliations:** 1 Division Integrative Risk Assessment, Data and Statistics, Austrian Agency for Health and Food Safety (AGES), Spargelfeldstraße 191, 1220 Vienna, Austria; tanja.tripolt@ages.at (T.T.); antonia.griesbacher@ages.at (A.G.); hans-peter.stueger@ages.at (H.P.S.); alexandra.wolf-spitzer@ages.at (A.W.-S.); 2 Department of Nutritional Sciences, University of Vienna, Althanstraße 14, 1090 Vienna, Austria; karl-heinz.wagner@univie.ac.at; 3 Care and Consumer Protection, Federal Ministry of Social Affairs, Health, Stubenring 1, 1010 Vienna, Austria; karin.schindler@meduniwien.ac.at (K.S.); adelheid.weber@gesundheitsministerium.gv.at (A.W.); 4 Division of Endocrinology and Metabolism, Department of Internal Medicine III, Medical University of Vienna, Währinger Gürtel 18-20, 1090 Vienna, Austria

**Keywords:** breastfeeding, exclusive breastfeeding, breastfeeding duration, exclusive breastfeeding duration, factors influencing, longitudinal, monitoring, infant formula

## Abstract

The WHO European Region has the lowest exclusive breastfeeding (EBF) rates at 6 months in the world. In Austria, 55.5% of infants are EBF at the age of one week, although breastfeeding initiation is 97.5%. The study was conducted in 2019/2020 and considered 1214 mothers of legal age, who received four online questionnaires during their child’s first year of life. The influence of different variables on total/exclusive breastfeeding duration were analysed by using a Cox model (Extension of the Cox Proportional Hazards Model) with time-dependent covariates. Multivariate analyses showed a significant influence of maternal BMI, lifestyle factors, such as smoking, and breastfeeding support on total breastfeeding duration. Remarkable differences in the median duration of any breastfeeding were found for breastfeeding support, where mothers breastfed twice as long. Support came primarily from hospital staff, the midwife and the partner. A higher monthly household net income, delivery in a baby-friendly certified hospital (BFH) and breastfeeding support were associated with a longer EBF duration. Obese mothers started feeding infant formula earlier and had a higher risk of early weaning. The results offer valuable insights into the importance of breastfeeding-friendly structures such as BFHs, a focus on breastfeeding aftercare and support of the mother to promote and protect breastfeeding.

## 1. Introduction

Breastfeeding is one of the first and most important health promotion measures in a child’s life. Breastfed children are less likely to become obese or overweight later in life, have a lower risk of non-communicable diseases such as diabetes, show lower rates of infections (e.g., middle ear infection) and perform better on intelligence tests [1,2]. It also has benefits for breastfeeding mothers, such as reduced risk of ovarian cancer and type 2 diabetes [3].

Globally, 44% of babies under 6 months of age are exclusively breastfed, whereas, at the age of 1 year, 68% of children are breastfed [4]. One of the global targets is to scale up exclusive breastfeeding to 70% at 6 months and total breastfeeding at 12 months to 80% by 2030 [4]. The WHO European Region has the lowest global exclusive breastfeeding rates at 6 months [5]. In Austria, the current rate of breastfeeding initiation is 97.5% but decreases to 40.8% after 1 year. At the age of 1 week, 55.5% of infants are exclusively breastfed and a first decrease is seen in the first 4 months. At the age of 17 weeks, 30.5% infants are exclusively breastfed [6].

The Austrian guidelines recommend exclusive breastfeeding for about six months. Complementary food can be introduced between the 17th and the 26th week of age, depending on the child’s individual development. Continued breastfeeding at the start of complementary feeding is advocated, as long as the mother and child wish to do so [7]. The minimum recommendation for the duration of exclusive breastfeeding, according to ESPGHAN, is 17 weeks [8].

Studies have repeatedly shown that exclusive breastfeeding rates can be increased through maternal support [9]. A systematic review highlights the multifactorial aspects of breastfeeding and a variety of patterns and drivers in different settings. It emphasises the importance of regularly monitoring breastfeeding patterns and important trends in breastfeeding as well as the need for multiple approaches to identify effective breastfeeding support [10]. Recently described barriers to exclusive breastfeeding are caesarean section, lack of knowledge regarding breastfeeding, awareness of an insufficient amount of breast milk, negative influence from society and family, returning to school or work, lack of privacy in public and private setting, among others [11].

The present study aims to increase the understanding of influencing factors (multivariate approach) determining breastfeeding duration and exclusive breastfeeding. This will support identifying possible entry points to promote and protect breastfeeding in Austria.

## 2. Materials and Methods

### 2.1. Data Collection and Study Design

The Austrian SUKIE-Study (SUKIE = Säuglings- und Kinderernährung) was conducted in 2019/2020. In this longitudinal survey, mothers were recruited in February/March 2019 in participating maternity wards all over Austria. From across the country, 64 (81%) maternity wards confirmed their voluntary participation. As approved by the Ethics Committees (Ref. 1303/2018, Ref. 90/2018, Ref. 2358, Ref. 30-438ex17/18, Ref. GS1-EK-4/555-2018, Ref. EKB13-18, Ref. EKB13-18), trained, multilingual fieldworkers and medical stuff were informed about the study and carried out the recruitment. Mothers who had not given birth in one of the participating hospitals were informed about the study, due to the support of midwives. Interested mothers could register themselves via the study homepage to enable participation also for those who had given birth at home. Mothers of newborns in intensive care were excluded. Participating mothers of legal age received an online questionnaire at four time points during their child’s first year of life (14 days, 4 months, 6 months and 12 months postpartum). Recruitment and documentation of participants was conducted in accordance with the STROBE statement [12]. The SUKIE-Study is enrolled at Clinical-Trials.gov (Trial registration: NCT04137796. Registration date 24 October 2019). Sample deviations from the population structure (age, education) were statistically taken into account. Therefore, the results can be considered as being representative. More details on data collection and study design have been previously published [6].

### 2.2. Questionnaire

Detailed questions about breastfeeding and infant nutrition, such as the first-time introduction of fluids and foods other than breast milk, reasons for breastfeeding, weaning, breastfeeding support (regarding the questions: Did you feel supported overall while breastfeeding? (Yes/No) By whom did you feel supported? (Partner, family, lactation consultant, breastfeeding group, midwife, hospital staff, gynecologist/doctor, open answer option)) as well as questions about the mother’s sociodemographic parameters and lifestyle were asked retrospectively [6]. The World Health Organization (WHO) definitions and “Infant and Young Child Feeding” indicators regarding breastfeeding [13,14] were used for better international comparability [8,15]. Total breastfeeding duration includes “exclusive breastfeeding”, “predominant breastfeeding” and “partial breastfeeding”. Exclusive breastfeeding means that the infant receives only breast milk (and, if required, medication, minerals, vitamins, oral rehydration solution). Predominant breastfeeding by definition uses breast milk as the main source, but also allows liquids such as water or water-based beverages. Partial breastfeeding/complementary feeding means that the child receives semi-solid, solid or soft foods in addition to breast milk. Further information about questionnaire development, pretests and indicators have been previously described [6].

### 2.3. Participants

A questionnaire was sent to over 1600 mothers. The follow-up rate varied between 61.7 and 77.6% depending on the time point of the survey. The detailed participant flow has already been shown [6]. After eligibility screening, 1214 mothers of legal age were included in the analysis. Mothers had a median age of 31 years (IQR 28; 35), 30.0% had a caesarean section. The distribution of education level and further sample characteristics have already been published [6].

### 2.4. Statistical Analysis

The statistical analyses were carried out with R Statistical Software (version 4.0.2) [16]. The influence of different variables on total/exclusive breastfeeding duration was investigated using a Cox model with time-dependent covariates, since the influence of certain variables changed over the observation period. Log Rank Tests were used to pre-select variables for statistical modeling. Statistically significant variables with a level of *p* < 0.2 were included in the model. Kaplan–Meier curves were chosen for the graphical representation of the influence of different variables on the breastfeeding duration (Extended Cox Proportional Hazards Model for Time-Dependent Variables [17]).

The following variables were used in the model: mother’s age (in categories) and education level (low/middle/high), mother’s country of birth (Austria/not Austria), occupation before pregnancy (working/not working before pregnancy), smoking during pregnancy (Yes = smoking in general and occasionally (less than 20 cigarettes/month)/No), delivery mode (vaginal delivery/caesarean section), breastfeeding support, regular physical activity/sports (as defined in the questionnaire as at least 2.5 h per week, e.g., cycling, fast walking or aerobics), mother herself was breastfed as an infant, infant sex and mother’s BMI (kg/m^2^) before pregnancy (underweight < 18.5/healthy weight 18.5–24.9/overweight 25.0–29.9/obese ≥ 30.0), family structure (single mother/with partner), monthly net household income (≤€2500/>€2500), delivery in a certified baby-friendly hospital, first birth.

#### 2.4.1. Variables for Breastfeeding Duration

The following variables were included in the Cox proportional hazards model: mother’s age and education level, mother’s country of birth, occupation before pregnancy, smoking during pregnancy, delivery mode, breastfeeding support, regular physical activity/sports, mother herself was breastfed as an infant, infant sex and mother’s BMI before pregnancy. The variables family structure, delivery in a certified baby-friendly hospital, first birth and monthly net household income, which did not reveal effects in the Kaplan–Meier survival curves, were excluded from the model.

#### 2.4.2. Variables for Exclusive Breastfeeding Duration

The following variables were included in the model of exclusive breastfeeding:

Mother’s age and education level, mother’s country of birth, monthly net household income, occupation before pregnancy, smoking during pregnancy, delivery in a certified baby-friendly hospital, breastfeeding support, regular physical activity/sports, mother herself was breastfed as an infant, first birth, delivery mode, infant sex and mother’s BMI before pregnancy. The variable family structure was excluded, due to no apparent effects in the Kaplan–Meier graphs and no significant log rank test.

#### 2.4.3. Model Subdivisions

Maternity leave is regulated by Austrian law and becomes obligatory at eight weeks before the expected date of birth and ends eight weeks after delivery [18]. According to the Austrian recommendations, complementary food can be introduced at 17 weeks of life, depending on the individual child’s development [7]. However, as a result, children who receive complementary food no longer belong to the category of “exclusive breastfeeding”. To account for these circumstances in the model, the following subdivisions were performed:

In terms of the total breastfeeding duration, the model was divided into two groups with a cutoff point at the age of eight weeks. This was also supported by the graphical analyses, which demonstrated a significant decrease in total breastfeeding duration seen after about eight weeks. Therefore, two models (0–8 weeks and 9–52 weeks after birth) were calculated and thus the time dependence was taken into account.

For calculations with regard to factors influencing exclusive breastfeeding duration, the model was divided into 3 groups: 0–1 week, 2–8 weeks and 9–17 weeks postpartum. The first week mainly concerns hospitalization; 8 weeks after delivery maternity protection is over and from the 17th week onwards, the recommendations could lead to cessation of exclusive breastfeeding.

## 3. Results

### 3.1. Sample Characteristics

The results of the SUKIE-Study showed that 97.5% of mothers had ever breastfed, as well as showing a decrease in breastfeeding after one year postpartum (Table 1). A descriptive evaluation of the prevalence of breastfeeding mothers at the different time points can be seen in Table 1. Mothers who returned to work within the first year after delivery were more likely not to breastfeed at 12 months (any breastfeeding at 12 months 31.1%, 95% CI: 23.2–39.0%) (not breastfeeding at 12 months—68.9%, 95% CI: 61.0–76.8%).

Almost every third child (30.5%) was exclusively breastfed four months (completed 17th week) after birth. Descriptive statistics about mothers exclusively breastfeeding four months after delivery are shown in Table 2.

### 3.2. Factors Influencing Total Breastfeeding Duration

**Figure 1:**
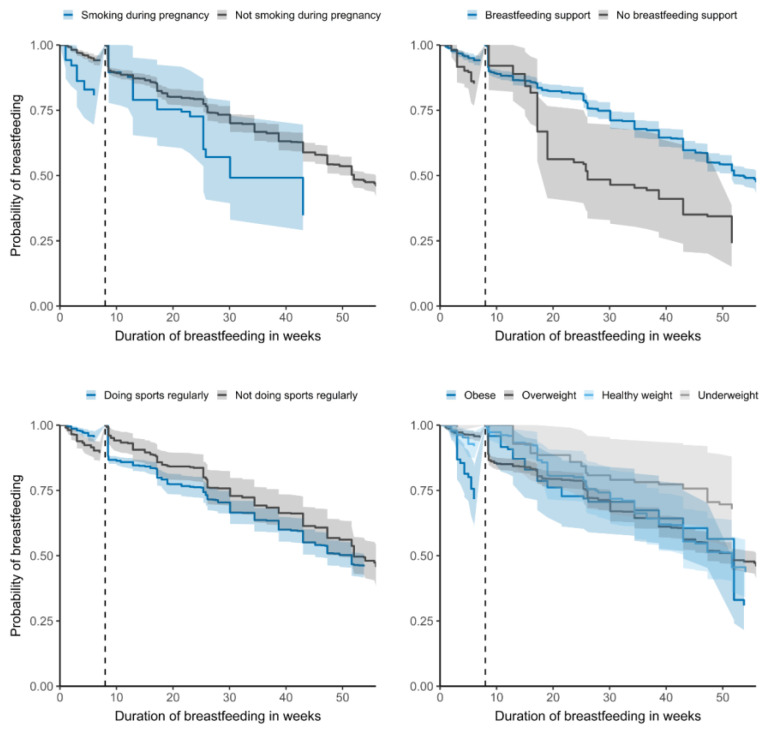
Kaplan–Meier survival curves for influencing factors on breastfeeding duration (*n* = 822).

Figure 1 shows Kaplan–Meier survival curves for factors smoking, sports, breastfeeding support and mother´s BMI. Multivariate analyses showed that the following factors influenced total breastfeeding duration significantly: maternal BMI, lifestyle factors, such as smoking or physical activity/sports, and breastfeeding support (Table 3). In terms of total breastfeeding duration, obese mothers were three times more likely to wean in the first eight weeks. Differences in the median total breastfeeding duration (in weeks) were found for smoking during pregnancy (median smoking: 25.4; 95% CI: 17.2–43.0) (median not smoking: 51.6; 95% CI: 47.3–53.8) and breastfeeding support (median support: 51.6; 95% CI: 47.3–55.6) (median no support: 25.4; 95% CI: 17.2–38.7).

Mothers indicated that breastfeeding support came primarily from hospital staff (74.2%; 95% CI: 67.7–80.7%), the midwife (69.5%; 95% CI: 66.4–72.6%) and the partner (64.5%; 95% CI:61.2–67.8%) followed by the family (30.9%; 95% CI: 27.8–34.0%), lactation consultant (15.6%; 95% CI: 13.3–17.6%), gynecologist (6.5%; 95% CI: 0.6–12.4%), breastfeeding group (2.0%; 95% CI: 1.0–2.9%) and others (1.8%; 95% CI: 0.9–2.7%). Multiple answers were possible for this question.

The reasons mentioned why mothers started to breastfeed their baby were: child health (93.5%; 95% CI: 91.8–95.1%), natural diet for the child (92.6%; 95% CI: 90.9–94.4%), supports mother–child bonding (80.2%; 95% CI: 75.1–85.3%), prevents child’s allergies (63.1%; 95% CI: 55.7–70.4%), it is practical (47.1%; 95% CI: 44.5–49.7%), helps with weight loss/involution (44.1%; 95% CI: 36.8–51.4%), brings joy (43.3; 95% CI: 37.6–49.1%), cheaper than infant formula (36.9%; 95% CI: 31.7–42.1%), health benefits for mother (35.4%; 95% CI: 29.7–41.2%), breastfeeding success with previous children (20.6%; 95% CI: 18.5–22.7%), was motivated by other people (10.5%; 95% CI: 8.8–12.2%), other reasons (0.9%; 95% CI: 0.3–1.6%). For this question, multiple answers were possible.

### 3.3. Factors Influencing Exclusive Breastfeeding Duration

**Figure 2:**
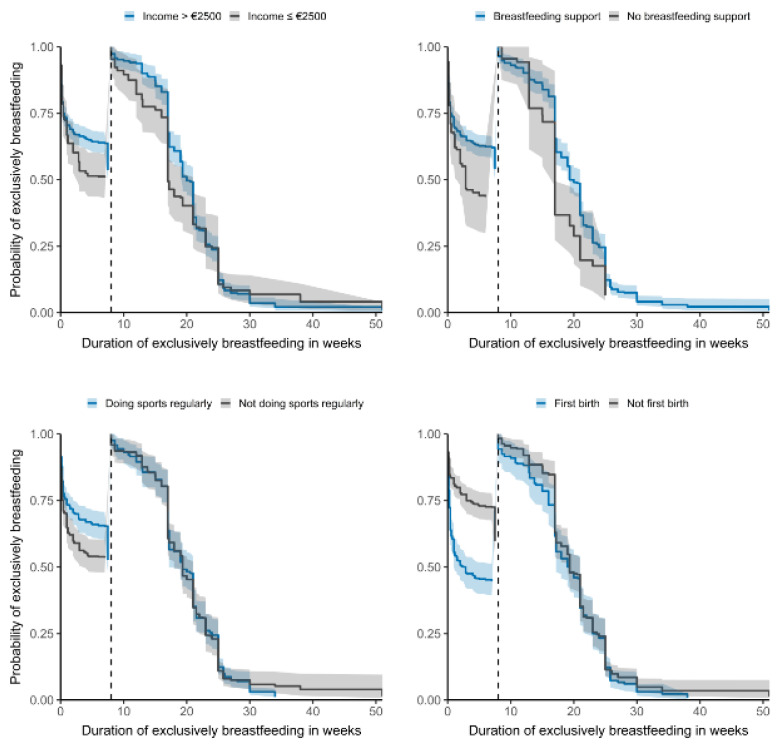
Kaplan–Meier survival curves for influencing factors on exclusively breastfeeding duration (*n* = 728).

The results showed that mothers with obesity started feeding infant formula earlier, especially in the first week after birth (Table 4). The median of exclusive breastfeeding duration in weeks differed depending on the mothers´ BMI (median underweight: 17.2; 95% CI: 15.0–21.0) (median healthy weight: 17.0; 95% CI: 13.0–17.2) (median overweight: 8.8; 95% CI: 2.1–17.0) (median obesity: 0.4; 95% CI: 0.3–4.3). Other significant factors influencing the median duration (in weeks) on exclusive breastfeeding are: breastfeeding support (16; 95% CI: 12.9–17.0), no support (2; 95% CI: 0.6–8.6), income <€2500 (6; 95% CI: 2.0–12.0), income >€2500 (17; 95% CI: 12.9–17.0) delivery in a baby-friendly hospital (17.2; 95% CI: 15.0–20.0), delivery not in a baby-friendly hospital (12.9; 95% CI: 8.6–16.0), first birth (5.5; 95% CI: 1.7–12.0), not first birth (17.0; 95% CI: 17.0–19), vaginal birth (16; 95% CI: 13.8–17.0), cesarean section (4; 95% CI: 1.0–12.0). Kaplan–Meier survival curves for income, breastfeeding support, sports and parity on exclusively breastfeeding duration are seen in Figure 2.

## 4. Discussion

In Austria, various measures and institutions for breastfeeding promotion exist, including the baby-friendly hospital initiative (BFHI) [19], associations of lactation consultants [20], a council of midwives [21], maternity leave policy [18], nursing leave at work [22], and the Early Childhood Interventions Program [23] to support vulnerable mothers. Nevertheless, the results of the SUKIE-Study show necessary improvements of breastfeeding duration and of the prevalence of exclusive breastfeeding. Other liquids than breastmilk, such as infant formula, water or tea, are introduced very early. Half of the infants receive formula for the first time within the first three days of life [6]. Regular monitoring of breastfeeding prevalence as well as influencing factors are an essential basis to identify vulnerable groups who are at risk of early weaning, which policymakers can enact contemporaneously [24].

The results show that mothers initiated breastfeeding primarily due to infant health, natural nutrition and the supportive aspect of mother–infant bonding. A caesarean section is associated with a higher risk of formula feeding in the first week. For future studies, it may be useful to distinguish between planned and emergency caesarean section. A planned caesarean section in particular is associated with lower breastfeeding rates. However, the type of delivery has no influence on breastfeeding prevalence at 6 months [25]. This reinforces the fact that the initiation is especially important for a sustainable continuation of breastfeeding. This is also seen for the factor parity. The Kaplan–Meier curve shows clearly this effect in first-time mothers. The Cox regression specifies significantly that feeding formula in the first week of life is twice as risky in first-time mothers. However, this effect disappeared after the second week.

Studies indicate that there is an association between the exposures of BFHI steps and the presumption of breastfeeding-improved outcomes [26]. Early skin-to-skin contact, as one of the BFHI steps, is known to have beneficial effects on breastfeeding [27,28,29]. The analyses show that the delivery in a certified baby-friendly hospital is associated with a significantly longer exclusive breastfeeding duration in the first week of life. Mothers who gave birth in a certified hospital have a 41% lower risk of weaning in the first week. Implementation of the WHO and UNICEF Ten Steps to successful breastfeeding [30] in the baby-friendly hospital initiative has a positive impact on breastfeeding outcomes [26], although more good-quality controlled trials are required [31]. Currently, in Austria, there exist 12 certified baby-friendly hospitals [19], because the successful implementation of baby-friendly hospital certification seems to depend on a number of barriers. A complex interplay of multiple factors as well as a challenge for the working routine of healthcare professionals would require structural changes but also continuous support efforts in Austria [32].

Particularly beneficial in terms of exclusive breastfeeding duration from the second week onwards is breastfeeding support. If the breastfeeding woman receives support, a significantly lower risk (65–69%, *p* < 0.01) of introducing infant formula between 2 and 17 weeks after birth is seen. Breastfeeding support also had a significant impact on the total breastfeeding duration in both time groups (*p* < 0.01). Mothers who feel supported have a 60% lower risk of weaning within the first eight weeks. These results are consistent with the outcome of the WHO and UNICEF Step 10, care at discharge [30] including community support, which is important for long-term sustainability of breastfeeding [26]. A review underlines these findings, that additional support by laypersons and professionals has a positive impact on breastfeeding rates [9]. Effective support options should be offered regularly and as standard by trained staff during prenatal care or postpartum. A set schedule of support services makes it predictable for mothers when they will receive support. Offerings should be tailored to the needs of the population and to the setting [9]. Kronborg and Væth recently published a screening tool to assess mothers at risk of early exclusive breastfeeding cessation before 17 weeks. This easy-to-use tool could be helpful to check who needs additional support during breastfeeding initiation [33]. Furthermore, community-based peer support (e.g., trained members of the community) is a promising approach for increasing the exclusive breastfeeding duration [11,34] and has a positive impact on breastfeeding outcomes by both professionals and laypersons [9]. The current results confirm that mothers receive breastfeeding support mainly from medical staff, the midwife and the partner. Trained peer counsellors could be implemented at healthcare facilities to counsel mothers [11]. Increased education on breastfeeding (e.g., how to cope with challenges, strategies to establish breastfeeding, pumping routines) and support during pregnancy improves initiation and continuation of breastfeeding [29]. The median total breastfeeding duration shows that mothers who are supported breastfeed twice as long as mothers who are not supported. The same remarkable differences are identified for smoking during pregnancy. A significant difference between smokers and non-smokers, from 9 to 52 weeks postpartum, have been observed. Women who smoked during pregnancy have a significant higher risk for early weaning. Smoking during pregnancy and lactation can affect milk production, and breast milk can contain secreted chemicals [35]. Nicotine has decreasing effects on the prolactin concentration in the blood and decreases milk production [36,37,38]. Smoking during breastfeeding increases the risk of respiratory diseases and allergies for the child [39]. In Europe, the percentage of mothers smoking during pregnancy is high. In 2015, Austria reported that 12.5% of mothers smoked during the third trimester [40]. There should be a focus on women with lower education, multiparous, with a lower socioeconomic status and single mothers who are at increased risk due to failure to quit smoking [41].

Likewise, the BMI of the mother is an important influencing variable on the total duration of breastfeeding. Obese mothers show a statistically significant three times higher risk of weaning in the first eight weeks. Concerning exclusive breastfeeding, especially in the first week after birth, obese mothers start formula feeding earlier. This effect increases in week two to eight. Obese mothers have a more than doubled risk of introducing infant formula. These outcomes are consistent with other studies and show again how important it is to offer guidance and counselling to obese women such as assistance for breastfeeding and additional education from conception onwards to maximise the success of breastfeeding [42,43]. Qualitative studies describe the additional difficulties obese women experience, for example, challenges with positioning their infant or latching, issues of body image when breastfeeding in public and physical challenge of large breasts [44,45]. Supportive measures for breastfeeding promotion should be developed together with the target group of obese women and should already be offered prenatally and continue postpartum [42,43,44,45].

Breastfeeding surveys in Europe show that a low education level is associated with a lower initiation of breastfeeding and earlier weaning [46]. Breastfeeding in high-income countries is more common in settings with a better socioeconomic level [47]. The results of the SUKIE-Study show that a higher income from the second week on is significantly associated with a longer exclusive breastfeeding duration. A monthly household net income below € 2500 shows an approximately 80% higher risk of introducing infant formula. Breastfeeding maintenance up to 12 months and longer is associated with factors such as older age of the mother, more previous children and a higher education level [48]. Maternity leave is fundamental to promote and support breastfeeding. Workplace interventions help to prevent early introduction of infant formula and increase breastfeeding duration [25]. Although Austria has a well-established maternity leave system, which includes maternity benefits, parental leave for up to two years after birth and an entitlement to “nursing time of 45 min” every eight hours upon return to work [22], descriptive analyses showed that mothers who returned to work within the first year postpartum were more likely not to breastfeed at 12 months. Further data about offered interventions in companies and the use of nursing leave during working days could bring more insights into the need for a breastfeeding-friendly environment at work and a possible entry point for a longer total breastfeeding duration.

### Strengths and Limitations of the Study

One of the strengths of the SUKIE-Study is the high response rate of the mothers, due to a sensitive approach during recruitment. Detailed questions and control questions about the first-time introduction of liquids other than breastmilk made it possible to avoid socially desirable answers and facilitated a precise allocation into the different breastfeeding categories. The subdivision into different time groups for the multivariate approach was important, since the Kaplan–Meyer-curves show that some of the effects of the influencing factors could only be seen in the first weeks after birth. The best example is the influencing factor physical activity. A trend reversal between the two time groups was seen clearly. In the first eight weeks after delivery there was a trend towards a longer breastfeeding period for female athletes. From 9 to 52 weeks, mothers who performed regular physical activity weaned significantly earlier. This shows that the subdivision into different time groups provides information about which factors are important in which phases of the breastfeeding period after birth. Similar to other studies, a higher proportion of well-educated mothers took part. The underrepresentation of mothers with a lower educational background was taken into account during the analyses to ensure representativeness [6]. Investigations within several settings, such as a combination of health system interventions and community interventions, show the largest effects on breastfeeding outcomes [10]. Another factor not investigated in this study is self-efficacy. Especially in the first month postpartum, a low self-efficacy seems to affect exclusive breastfeeding duration so that psychosocial aspects are important to include in effective breastfeeding support [49]. These aspects should be included in further research.

## 5. Conclusions

Multivariate analyses of the SUKIE-Study provide valuable insights into determinants of total breastfeeding duration as well as of exclusive breastfeeding. Breastfeeding-friendly structures such as baby-friendly hospitals, the promotion of exclusive breastfeeding at the beginning at all levels, a focus on breastfeeding aftercare as well as structures for long-term support of the mother are very promising approaches to promote and protect breastfeeding. A special focus should be on high-risk populations such as obese mothers, mothers who smoked during pregnancy and low-income families. The current results confirm previous findings and give an important and more precise insight into which factors are important for promoting breastfeeding in Austria.

## Figures and Tables

**Table 1:** Prevalence of mothers who had ever breastfed and breastfeeding mothers at 6 and 12 months after delivery separated by age, country of birth and education level in % of population (*n* = 1.214).

	Ever Breastfed		Any BF at 6 Months		Any BF at 12 Months	
	%	95% CI	%	95% CI	%	95% CI
Total	97.5	96.5–98.6	64.1	55.8–72.5	40.8	31.8–49.7
Age (years)						
18–24	98.2	95.7–100.0	56.2	47.0–65.5	23.5	12.6–34.4
25–29	96.8	94.6–99.0	56.8	38.9–74.7	35.5	17.2–53.8
30–34	97.4	95.6–99.2	64.1	46.8–81.4	41.3	23.3–59.2
35–39	98.1	96.4–99.9	80.3	75.4–85.2	56.0	50.1–61.8
>40	100.0	93.6–100.0	81.9	70.0–93.8	61.2	46.4–76.0
Country of birth						
Austria	97.2	95.9–98.5	60.0	50.9–69.1	35.7	26.4–44.9
other country	98.5	97.2–99.7	78.6	71.0–86.3	57.4	45.3–69.4
Education level						
low	94.9	89.2–100.0	50.7	31.5–69.9	26.0	10.4–41.6
middle	97.6	96.5–98.7	60.2	49.0–71.3	40.0	27.9–52.2
high	99.0	98.2–99.9	83.7	80.3–87.0	49.3	44.7–53.9

BF: Breastfeeding; CI: confidence intervals.

**Table 2:** Prevalence of exclusively breastfeeding (EBF) mothers, not exclusively breastfeeding mothers and not breastfeeding (BF) mothers at completed 4 months after delivery separated by age, country of birth and education level in % of population (*n* = 1.214).

	EBF		Not EBF		Not BF	
	%	95% CI	%	95% CI	%	95% CI
Total	30.5	28.0–33.1	46.9	41.1–52.6	22.6	17.0–28.3
Age (years)						
18–24	16.0	8.3–23.7	47.9	39.1–56.7	36.1	26.7–45.6
25–29	27.6	22.9–32.3	40.5	23.1–58.0	31.9	14.5–49.2
30–34	35.4	31.3–39.4	49.5	45.5–53.6	15.1	11.6–18.6
35–39	35.4	29.5–41.3	52.5	46.8–58.2	12.1	8.0–16.2
>40	44.5	30.2–58.8	42.7	28.0–57.4	12.8	1.8–23.7
Country of birth						
Austria	32.1	28.5–35.7	43.5	39.4–47.6	24.4	18.7–30.1
other country	25.0	16.8–33.1	58.5	47.2–69.8	16.5	10.1–22.9
Education level						
low	35.1	17.9–52.2	32.4	16.1–48.7	32.5	15.9–49.2
middle	24.3	21.5–27.0	50.8	43.2–58.4	24.9	17.4–32.5
high	51.9	47.4–56.3	37.9	33.6–42.2	10.2	7.6–12.9

**Table 3:** Cox regression of factors influencing shorter breastfeeding duration considered separately for 2 different age ranges of infants (*n* = 822).

	Infant Age in Completed Weeks					
	0–8			9–52		
	HR	95% CI	*p*-Value	HR	95% CI	*p*-Value
Mother not born in Austria	1.00			1.00		
Mother born in Austria	0.94	0.48–1.82	0.85	1.23	0.92–1.65	0.17
Mother not working before pregnancy	1.00			1.00		
Mother working before pregnancy	1.42	0.63–3.24	0.40	1.17	0.85–1.62	0.34
Mother not smoking during pregnancy	1.00			1.00		
Mother smoking during pregnancy	1.87	0.85–4.11	0.12	1.81	1.15–2.87	**0.01**
Vaginal delivery	1.00			1.00		
Caesarean section	1.57	0.91–2.71	0.11	1.20	0.94–1.53	0.15
No breastfeeding support	1.00			1.00		
Breastfeeding support	0.40	0.21–0.75	**<0.01**	0.54	0.37–0.78	**<0.01**
Mother not practising sports regularly	1.00			1.00		
Mother practising sports regularly	0.67	0.40–1.12	0.13	1.27	1.02–1.57	**0.03**
Mother not breastfed as infant	1.00			1.00		
Mother breastfed as infant	0.62	0.37–1.02	0.06	0.88	0.71–1.10	0.26
Infant sex: girl	1.00			1.00		
Infant sex: boy	0.87	0.52–1.44	0.59	0.86	0.70–1.05	0.13
BMI mother: healthy weight	1.00			1.00		
BMI mother: underweight	0.29	0.04–2.15	0.23	0.73	0.45–1.16	0.18
BMI mother: overweight	0.90	0.45–1.80	0.77	0.98	0.76–1.27	0.89
BMI mother: obese	3.17	1.69–5.93	**<0.01**	1.35	0.89–2.06	0.16

HR = hazard ratio, CI = confidence interval, BMI = body mass index, Bold = statistically significant. Adjusted for mother’s age and mother’s education level.

**Table 4:** Cox- regression of factors influencing exclusively breastfeeding duration considered separately for 3 different age ranges of infants (*n* = 728).

	Infant Age in Completed Weeks								
	1 Week of Age			2–8 Weeks of Age			9–17 Weeks of Age		
	HR	95% CI	*p*-Value	HR	95% CI	*p*-Value	HR	95% CI	*p*-Value
Mother not born in Austria	1.00			1.00			1.00		
Mother born in Austria	1.11	0.79–1.56	0.55	0.72	0.39–1.31	0.28	0.79	0.48–1.30	0.36
Monthly net household income >€2500	1.00			1.00			1.00		
≤€2500	1.05	0.76–1.44	0.79	1.86	1.06–3.25	**0.03**	1.71	1.06–2.75	**0.03**
Mother not working before pregnancy	1.00			1.00			1.00		
Mother working before pregnancy	1.02	0.66–1.59	0.93	1.19	0.54–2.59	0.67	1.68	0.85–3.32	0.14
Mother not smoking during pregnancy	1.00			1.00			1.00		
Mother smoking during pregnancy	1.07	0.64–1.80	0.80	0.43	0.10–1.83	0.25	1.92	0.87–4.21	0.11
Delivery not in BFH	1.00			1.00			1.00		
Delivery in BFH	0.59	0.40–0.89	**0.01**	0.83	0.42–1.63	0.58	0.79	0.44–1.42	0.44
No breastfeeding support	1.00			1.00			1.00		
Breastfeeding support	0.99	0.65–1.50	0.95	0.31	0.16–0.59	**<0.01**	0.35	0.19–0.67	**<0.01**
Mother not practising sports regularly	1.00			1.00			1.00		
Mother practising sports regularly	0.79	0.61–1.02	0.07	0.61	0.37–1.01	0.05	0.94	0.63–1.42	0.78
Mother not breastfed as infant	1.00			1.00			1.00		
Mother breastfed as infant	0.88	0.68–1.14	0.34	0.73	0.43–1.21	0.22	1.03	0.66–1.60	0.92
Not first birth	1.00			1.00			1.00		
First birth	2.02	1.53–2.67	**<0.01**	1.02	0.60–1.73	0.95	1.02	0.66–1.58	0.93
Vaginal birth	1.00			1.00			1.00		
Caesarean section	1.38	1.04–1.83	**0.03**	1.61	0.93–2.79	0.09	0.65	0.36–1.16	0.15
Infant sex: girl	1.00			1.00			1.00		
Infant sex: boy	1.05	0.82–1.34	0.71	0.67	0.41–1.10	0.12	0.97	0.65–1.45	0.88
BMI mother: healthy weight	1.00			1.00			1.00		
BMI mother: underweight	0.72	0.40–1.31	0.29	0.15	0.02–1.12	0.06	1.37	0.69–2.71	0.37
BMI mother: overweight	1.20	0.88–1.63	0.26	1.19	0.65–2.17	0.57	1.41	0.87–2.27	0.16
BMI mother: obese	1.67	1.11–2.51	**0.01**	2.34	1.03–5.31	**0.04**	1.00	0.35–2.89	0.99

HR = hazard ratio, CI = confidence interval, BFH = baby-friendly hospital, BMI = body mass index, Bold = statistically significant. Adjusted for mother’s age and mother’s education level.

## Data Availability

The data presented in this study are available on request from the corresponding author.
